# Editorial: The multifaceted role of cholangiocytes in neoplastic and non-neoplastic cholangiopathies: from bench to bedside

**DOI:** 10.3389/fgstr.2026.1874974

**Published:** 2026-07-15

**Authors:** Alessandro Gambella, Michele Pinon

**Affiliations:** 1 Pathology Unit, Department of Surgical Sciences and Integrated Diagnostics (DISC), University of Genoa, Genoa, Italy; 2 Pathology Unit, Istituto di Ricovero e Cura a Carattere Scientifico (IRCCS) Azienda Ospedaliera Metropolitana (IRCCS AOM), Genoa, Italy; 3 Pediatric Gastroenterology Unit, Regina Margherita Children’s Hospital, Azienda Ospedaliera-Universitaria Città della Salute e della Scienza, Turin, Italy

**Keywords:** cholangiocyte biology, cholangiocytes, genetic cholangiopathy, immune-metabolic crosstalk, next-generation pathology

Since the first description of the canals of Hering and the detailed characterization of biliary tract anatomy and hepatic epithelial cells (1–3), as well as the introduction and consolidation of the term “cholangiocyte” (4, 5), a central concept has emerged in hepatobiliary pathobiology: biliary epithelial cells do not simply line the bile ducts. Rather, they are dynamic, highly specialized cells that orchestrate bile secretion, epithelial repair, ductular reaction, immune signaling, fibrogenesis, and neoplastic transformation.

Under physiological conditions, cholangiocytes contribute to the maintenance of biliary homeostasis through finely regulated secretory, absorptive, and immunomodulatory activities (6). In disease states, however, this same plasticity may become maladaptive, triggering chronic inflammation, fibrosis, ductopenia, stricturing disease, and carcinogenesis. This view is reinforced by recent work highlighting cholangiocyte heterogeneity, senescence-associated secretory programs, portal fibroblast activation and epithelial plasticity, and immune-metabolic crosstalk as central mechanisms of chronic biliary injury leading to fibrosis and cancer development (7–9). In particular, genetic and developmental cholangiopathies, such as Alagille syndrome, progressive familial intrahepatic cholestasis, cystic fibrosis-related cholangiopathy, and bile acid synthesis defects, represent a particularly informative area within this spectrum because they demonstrate how altered biliary development, epithelial differentiation, transporter function, and ductal architecture can produce lifelong cholestatic phenotypes, requiring genotype-informed follow-up in both pediatric and adult cholestatic liver disease (10, 11).

Our Research Topic, “The multifaceted role of cholangiocytes in neoplastic and non-neoplastic cholangiopathies: from bench to bedside,” was developed to integrate these biological and clinical perspectives, and the articles collected emphasize this translational continuum: from prognostic modeling in autoimmune cholestatic liver disease to diagnostic pitfalls in immune-mediated pancreaticobiliary disorders, interventional palliation of malignant obstruction, and rare hepatobiliary neoplasia. Together, they highlight an additional key concept: accurate phenotyping of cholangiocytes and biliary disease complexity requires a cross-platform approach integrating clinical presentation, biochemical signatures, advanced imaging, pathology, immunology, and molecular analyses.

In primary biliary cholangitis (PBC), chronic immune-mediated injury of small intrahepatic bile ducts remains paradigmatic. Liu et al. developed a clinically accessible prognostic nomogram for 5-year survival in 315 patients with PBC treated with ursodeoxycholic acid. By integrating age, neutrophil-to-lymphocyte ratio, and total bilirubin, the model achieved robust discrimination in both training and validation cohorts, with AUC values of 0.7251 and 0.7721, respectively, and stratified patients into prognostically distinct risk groups (Liu et al.). This work is particularly relevant because it harmonizes cholestasis, systemic inflammation, and patient aging into a practical tool for daily clinical use.

Two contributions highlight the diagnostic complexity of immune-mediated pancreatobiliary disease. Guglielmo et al. describe a young patient with obstructive jaundice due to type 2 autoimmune pancreatitis, later associated with Crohn’s disease and autoimmune hemolytic anemia. This case emphasizes that jaundice in young adults, even when raising concern for malignancy, may instead reflect a non-neoplastic and, in this instance, steroid-responsive immune-mediated disorder. The subsequent development of Crohn’s disease and hemolytic anemia symptoms further demonstrates that biliary presentations may be the first manifestation of a broader immune-related disorder, requiring dedicated follow-up beyond the initial resolution of cholestasis.

Yang et al. report a case in which tissue characterization was pivotal. They address IgG4-related sclerosing cholangitis, a disease whose clinical and radiologic phenotype may closely resemble perihilar cholangiocarcinoma. Their case is instructive because definitive diagnosis required histopathology and immunohistochemistry rather than clinical presentation and serology alone; serum IgG4 may be normal or unavailable early in the disease course; and tissue-based diagnostic confirmation can be essential in cases of indeterminate biliary strictures.

The neoplastic and palliative dimensions of cholangiocyte-related disease are addressed by Bock et al., who systematically reviewed percutaneous uncovered metallic biliary stents for malignant obstruction. Across 1,453 patients, technical success was high at 97.7%, stent occlusion occurred in 13.5%, and 6.6% required reintervention to maintain patency. However, complications occurred in 19.1% of patients, with pancreatitis, cholangitis, and bleeding being the most frequent events. These data are highly relevant to clinical decision-making in advanced biliary malignancy, where palliation must balance technical feasibility, symptom relief, procedural risk, expected survival, and quality of life.

Finally, Wang et al. report a rare synchronous double primary malignancy: combined hepatocellular–cholangiocarcinoma with gallbladder adenocarcinoma. As the field of primary hepatic malignancies is undergoing a much-needed conceptual and diagnostic refinement, this report stresses the importance of accurate radiologic–pathologic correlation in distinguishing independent primary tumors from metastatic disease. Pathologic analysis of the surgical sample demonstrated a combined hepatocellular–cholangiocarcinoma and a separate gallbladder adenocarcinoma, supporting the diagnosis of synchronous double primary hepatobiliary cancers.

Overall, the articles collected in our Research Topic show that cholangiopathies occupy a biologically continuous and clinically heterogeneous spectrum. At one end are immune-mediated disorders in which cholangiocytes are both targets and amplifiers of inflammation; at the other are malignancies in which neoplastic biliary epithelial cells, chronic injury, and immune-metabolic crosstalk create a complex tumor and peritumoral microenvironment that still requires much-needed dedicated investigation. Indeed, future work should further define the morpho-molecular signatures characterizing cholangiocyte activation, senescence, immune-metabolic crosstalk, and malignant transformation, while translating these insights into biomarkers, targeted therapies, and individualized care pathways.

In conclusion, the contributions to this Research Topic reaffirm the crucial role of cholangiocytes by bridging bench-derived concepts with real-world diagnostic and therapeutic challenges. We believe this Research Topic can offer a clinically meaningful framework for understanding biliary disease and improving outcomes for patients across the cholangiopathy spectrum.

On a personal note, we would like to sincerely thank all authors who contributed to this Research Topic, as well as the reviewers whose time, expertise, and dedication made this collection possible.
